# Perceived Harm of Vaping Relative to Smoking and Associations With Subsequent Smoking and Vaping Behaviors Among Young Adults: Evidence From a UK Cohort Study

**DOI:** 10.1093/ntr/ntaf018

**Published:** 2025-02-14

**Authors:** Katherine East, Eve Taylor, Ann McNeill, Ioannis Bakolis, Amy E Taylor, Olivia M Maynard, Marcus R Munafò, Jasmine Khouja

**Affiliations:** Department of Primary Care and Public Health, Brighton and Sussex Medical School, Brighton, UK; National Addiction Centre, Institute of Psychiatry, Psychology and Neuroscience, King’s College London, London, UK; National Addiction Centre, Institute of Psychiatry, Psychology and Neuroscience, King’s College London, London, UK; Department of Behavioural Science and Health, University College London, London, UK; National Addiction Centre, Institute of Psychiatry, Psychology and Neuroscience, King’s College London, London, UK; Department of Biostatistics & Health Informatics, Institute of Psychiatry, Psychology and Neuroscience, King’s College London, London, UK; Population Health Sciences, Bristol Medical School, University of Bristol, Bristol, UK; MRC Integrative Epidemiology Unit, University of Bristol, Bristol, UK; MRC Integrative Epidemiology Unit, University of Bristol, Bristol, UK; School of Psychological Science, University of Bristol, Bristol, UK; MRC Integrative Epidemiology Unit, University of Bristol, Bristol, UK; School of Psychological Science, University of Bristol, Bristol, UK; MRC Integrative Epidemiology Unit, University of Bristol, Bristol, UK; School of Psychological Science, University of Bristol, Bristol, UK

## Abstract

**Introduction:**

There is a lack of evidence on whether vaping harm perceptions can predict vaping and smoking behaviors among young adults in the United Kingdom. We aimed to assess whether the perceived harm of vaping relative to smoking is associated with subsequent changes in vaping and smoking behaviors in this population.

**Methods:**

Data were from the Avon Longitudinal Study of Parents and Children (ALSPAC), a prospective cohort study. Longitudinal associations were assessed between the perceived harm of vaping relative to smoking at baseline (approximately 24 years old; Nov’15–Aug’17) and the following smoking/vaping outcomes at follow-up (approximately 30 years old; May–Oct’22): (1) stopping smoking, (2) initiation of ever smoking and/or vaping, and (3) uptake of past 30-day smoking and/or vaping. Multinomial logistic regressions were used, adjusting for sociodemographic factors.

**Results:**

Among young adults who smoked but did not vape at baseline (*n* = 687), the perception that vaping is less harmful than smoking (vs. equally/more harmful, or don’t know) was associated with stopping smoking and now vaping at follow-up (adjusted Relative Risk Ratio (aRRR)=1.69, 95%CI = 1.02 to 2.81, *p* = .04). Initiation of ever smoking/vaping, or uptake of past 30-day smoking/vaping, were not common during the study period and there was little evidence that these outcomes were associated with relative vaping harm perceptions at baseline.

**Conclusions:**

Among young adults who smoke, perceiving vaping as less harmful than smoking was associated with switching from smoking to vaping six years later. Few young adults who did not smoke or vape initiated these behaviors during the study period.

**Implications:**

This is the first study in England to find that young adults who smoked and who accurately perceived vaping as less harmful than smoking were more likely to switch to vaping 6 years later. This is consistent with prior studies among adults and highlights the need for interventions to improve the pervasive misperceptions about vaping that are currently observed among young adults who smoke.

## Introduction

Vaping carries some risks but is less harmful than smoking and can help people quit smoking.^1–4^ However, misperceptions of vaping harms are increasing; in England in 2024, 85% of adults who smoked inaccurately perceived that vaping is equally or more harmful than smoking or did not know the relative harms, an increase from 59% in 2014.^5^

Evidence reviews suggest that vaping harm perceptions can influence vaping behaviors.^1,3^ Accurately perceiving vaping as less harmful than smoking can predict subsequent increases in vaping, including starting vaping, among young people and adults.^1,3^ Additionally, perceiving vaping as harmful is associated with not subsequently initiating vaping among young people (aged <18 years) and young adults (aged 18–35 years).^1,3^

There is less longitudinal evidence on whether vaping harm perceptions can influence *smoking* behaviors.^1,3^ The limited evidence that exists suggests that, among adults, accurately perceiving vaping as less harmful than smoking predicted quitting smoking while inaccurately perceiving vaping as equally or more harmful than smoking predicted relapse to smoking.^1,3^ The few studies in this area among young adults have all been conducted in the United States and found little evidence of associations.^1,3^ Little is known about whether the perceived harms of vaping compared with smoking can predict subsequent vaping and smoking behaviors among young adults in the United Kingdom.

This study therefore assessed whether perceived harm of vaping relative to smoking is associated with subsequent vaping and smoking behaviors among young adults. Specific aims are to assess whether perceived harm of vaping relative to smoking is longitudinally associated with: (1) stopping smoking, (2) initiation of ever smoking and/or vaping, and (3) uptake of past-30-day smoking and/or vaping.

## Methods

### Data Source

Data were from the Avon Longitudinal Study of Parents and Children (ALSPAC), a prospective cohort study from birth to young adulthood.^6–8^ Briefly, ALSPAC recruited pregnant women residing in Avon, UK, with expected delivery dates 1991–1992, with 15 447 pregnancies resulting in 15 658 fetuses. Of these, 14 901 children were alive at 1 year of age. Supplementary Figure S1 shows the sample selection; briefly, we included respondents who completed the vaping/smoking questionnaires at age 24+ (Nov’16–Aug’17; ages 24–26 years) or, where data were missing, age 23+ (Nov’15–Sep’16; ages 23–25 years), and age 30+ (May–Oct’22; ages 30–32 years). Ages 23+/24+ questionnaires were “baseline,” because this is when respondents were first asked about relative vaping harm perceptions. Age 30+ questionnaire was “follow-up.”

Study data were collected and managed using REDCap electronic data capture tools hosted at the University of Bristol.^9^ REDCap is a secure, web-based software platform designed to support data capture for research studies. Ethics approval was obtained from the ALSPAC Ethics and Law Committee and the Local Research Ethics Committees. Informed consent was obtained following the recommendations of the ALSPAC Ethics and Law Committee at the time. The study website contains details of all data available through a fully searchable data dictionary and variable search tool (www.bristol.ac.uk/alspac/researchers/our-data).

### Measures

Measures are detailed in the Supplementary Materials. Briefly, the exposure was the perception that vaping is less harmful than smoking vs. otherwise (equally/more harmful, or don’t know), consistent with prior work.^10,11^ Potential confounders were selected and coded to be consistent with prior ALSPAC work assessing smoking and vaping^12,13^: baseline questionnaire completed, sex-at-birth,^12^ race/ethnicity,^12^ age 22 unemployment status,^12^ mother/partner occupation,^13^ and mothers smoking during pregnancy^12^ (Table 1). Outcomes were:

**Table 1.** Sample Characteristics and Attrition Analyses.

	Retained in complete case sample (*n* = 3211)	Lost to follow-up (*n* = 1892)	Contrast of loss to follow-up vs. retained in sample	
	*n* (%)	*n* (%)	OR (95% CI)	*p* value
Sex assigned at birth				
Female	2201 (69%)	1050 (55%)	Ref	
Male	1010 (31%)	842 (45%)	1.75 (1.55–1.97)	<.01
Race/ethnicity				
White	2766 (86%)	1565 (83%)	Ref	
Racialized minorities	103 (3%)	78 (4%)	1.34 (0.99–1.81)	.06
Missing	342 (11%)	249 (13%)	1.29 (1.08–1.53)	.01
Unemployed/not in education, age 22				
No	2330 (73%)	882 (47%)	Ref	
Yes	150 (5%)	74 (4%)	1.30 (0.98–1.74)	.07
Missing	731 (23%)	936 (49%)	3.40 (3.00–3.83)	<.01
Mother/partner occupation				
Professional	508 (16%)	261 (14%)	Ref	
Managerial and technical	1191 (37%)	650 (34%)	1.06 (0.89–1.27)	.50
Skilled manual or nonmanual	791 (25%)	471 (25%)	1.16 (0.96–1.40)	.12
Semiskilled manual/unskilled	60 (2%)	44 (2%)	1.43 (0.94–2.17)	.09
Missing	661 (21%)	466 (25%)	1.37 (1.13–1.66)	<.01
Mother smoked during the first 3 months of pregnancy				
No	2488 (77%)	1356 (72%)	Ref	
Yes	454 (14%)	342 (18%)	1.38 (1.18–1.61)	<.01
Missing	269 (8%)	194 (10%)	1.32 (1.09–1.61)	.01
Baseline questionnaire completed				
23+	360 (11%)	513 (27%)	Ref	
24+	2851 (89%)	1379 (73%)	0.34 (0.29–0.39)	<.01
Baseline perceived vaping harm perceptions				
Vaping is less harmful than smoking (accurate)	1437 (45%)	757 (40%)	Ref	
Vaping is equally/more harmful than smoking, or don’t know	1774 (55%)	1135 (60%)	1.21 (1.08–1.36)	.001
Baseline ever smoking/vaping				
Neither	1196 (37%)	578 (31%)	Ref	
Only smoked	1106 (34%)	669 (35%)	1.25 (1.09–1.44)	<.01
Only vaped	59 (2%)	31 (2%)	1.50 (1.30–1.73)	<.01
Both smoked and vaped	845 (26%)	610 (32%)	1.09 (0.70–1.70)	.71
Missing	<5^1^ (<1%)	<5^1^ (<1%)	2.76 (0.62–12.39)	.18
Baseline past 30-day smoking/vaping				
Neither	2375 (74%)	1247 (66%)	Ref	
Only smoked	687 (21%)	516 (27%)	1.43 (1.25–1.63)	<.01
Only vaped	88 (3%)	78 (4%)	1.69 (1.24–2.31)	<.01
Both smoked and vaped	59 (2%)	48 (3%)	1.55 (1.05–2.28)	.03
Missing	<5^1^ (<1%)	<5^1^ (<1%)	2.86 (0.48–17.12)	.25

OR = unadjusted odds ratio; 95% CI = 95% confidence interval.

^1^Cell counts less than 5 (including zero) are reported as <5 in line with ALSPAC’s requirements https://www.bristol.ac.uk/media-library/sites/alspac/documents/alspac-publications-checklist.pdf.

1. Stopping smoking (including now vaping). Among those who only smoked (did not vape) in the past 30 days at baseline: (1-reference) past 30-day smoking only at follow-up (ie, still smoking); (2) no past 30-day smoking/vaping at follow-up (ie, stopped smoking, also not vaping); (3) past 30-day vaping only at follow-up (ie, stopped smoking and now vaping); (4) past 30-day vaping and smoking at follow-up (ie, still smoking and also now vaping). Categories 1–4 are mutually exclusive.
2. Initiating ever smoking and/or vaping. Among those who had never vaped or smoked at baseline: (1-reference) never smoked/vaped at follow-up (ie, abstained); (2) ever smoked only at follow-up (ie, tried smoking); (3) ever vaping only at follow-up (ie, tried vaping), (4) ever smoked and vaped at follow-up (ie, tried both). Categories 1–4 are mutually exclusive.
3. Uptake of past 30-day smoking and/or vaping. Among those who had not vaped or smoked in the past 30 days at baseline: (1-reference) no past 30-day smoking/vaping at follow-up (ie, no uptake of current smoking/vaping); (2) past 30-day smoking only at follow-up (ie, uptake of current smoking); (3) past 30-day vaping only at follow-up (ie, uptake of current vaping); (4) past 30-day smoking and vaping at follow-up (ie, uptake of both). Categories 1–4 are mutually exclusive.

### Missing Data and Attrition

Baseline data were from *n* = 5110 young adults who had valid data on vaping harm perceptions at age 24+ (*n* = 4237) or, where data were missing, age 23+ (*n* = 873). Of these, seven were excluded for missing data on sex-at-birth and 1892 were lost to follow-up due to not completing the questionnaire or having missing data at age 30+, leaving 3211 in the sample (Supplementary Figure 1). For covariates, missing data were coded as “missing” to maximize sample size, except sex-at-birth whereby few had missing data and so were excluded. Attrition was associated with the outcome, exposure, and covariates (Table 1) so likely missing not at random (MNAR)—under this assumption, multiple imputation might bias estimates and so we ran complete case analyses with *n* = 3211 respondents.^14,15^

### Analyses

Analyses were run using Stata v18. First, we reported the characteristics of respondents in the analytic sample (*n* = 3211) and compared them to respondents lost to follow-up (*n* = 1892) using unadjusted logistic regressions. Second, to assess whether vaping harm perceptions at baseline were associated with smoking/vaping outcomes, we ran unadjusted and adjusted multinomial logistic regressions. Third, because ~20% of the sample responded “don’t know” to the exposure, we ran sensitivity analyses with “don’t know” responses coded as a unique category to assess whether this impacted the results. Fourth, to assess whether vaping harm perceptions (exposure) were stable over time, we compared perceptions at baseline and follow-up using a McNemar’s χ2 test (see Supplementary Materials for details and results). *p* Values were not corrected for multiple comparisons because doing so can increase the probability of type II errors and increase the risk of interpretation errors.^16^ Instead, we report exact effect sizes, confidence intervals, and *p* values.

## Results

### Sample Characteristics

The complete case sample (*n* = 3211) comprised mainly females, those of white race/ethnicity, employed/in education/training at age 22, had a mother/their partner in a managerial/technical, or skilled manual/non-manual, occupation, and had a mother who did not smoke within the first 3 months of pregnancy (Table 1). Most held the misperception that vaping is equally/more harmful than smoking or did not know, and had neither smoked nor vaped in the past 30 days at baseline (Table 1). Attrition was more likely among respondents who had inaccurate vaping perceptions, smoked and/or vaped ever or in the past 30 days, were male, had a mother who smoked during the first 3 months of pregnancy and had missing data on covariates (Table 1).

### Associations Between Relative Vaping Harm Perceptions and Subsequent Vaping and Smoking Behaviors

#### Stopping Smoking (Including Now Vaping)

Among those who smoked but did not vape in the past 30 days at baseline (*n* = 687), 32% (*n* = 220) were still smoking and did not vape at follow-up, 37% (*n* = 253) had stopped smoking and were also not vaping, 14% (*n* = 93) had stopped smoking and were now vaping, and 18% (*n* = 121) were still smoking and now also vaping. Perceiving that vaping is less harmful than smoking (vs. equally/more harmful, or don’t know) was associated with an increased likelihood of stopping smoking and now vaping in adjusted analyses (Table 2). There was little evidence for any other differences.

**Table 2.** Associations Between Perceived Harm of Vaping Relative to Smoking at Baseline and Subsequent Smoking and Vaping at Follow-Up.

1) Stopping smoking (including now vaping) among those who had smoked in the past 30 days at baseline (n = 687):							
	Still smoking only (n = 220; ref)	Stopped smoking, also not vaping (n = 253)		Stopped smoking and now vaping (n = 93)		Still smoking and now vaping (n = 121)	
Perception that vaping is less harmful than smoking	*n* (%)	*n* (%)	Relative risk ratio (95% CI), *p*	*n* (%)	Relative risk ratio (95% CI), *p*	*n* (%)	Relative risk ratio (95% CI), *p*
No/don’t know	139 (35%)	139 (35%)	Ref	50 (13%)	Ref	68 (17%)	Ref
Yes	81 (28%)	114 (40%)	RRR = 1.41 (0.97–2.04), *p* = .07 aRRR=1.37 (0.94–2.01), *p* = .10	43 (15%)	RRR = 1.48 (0.90–2.41), *p* = .12 aRRR=1.69 (1.02–2.81), *p* = .04	53 (18%)	RRR = 1.34 (0.85–2.10), *p* = .21 aRRR = 1.31 (0.82–2.09), *p* = .26
2) Initiating ever smoking, vaping, or both among those who had never smoked nor vaped at baseline (*n* = 1198):							
	No initiation (*n* = 1051; ref)	Initiation of ever smoking only (*n* = 75)		Initiation of ever vaping only (*n* = 40)		Initiation of both (*n* = 32)	
Perception that vaping is less harmful than smoking	*n* (%)	*n* (%)	Relative risk ratio (95% CI), p	*n* (%)	Relative risk ratio (95% CI), p	*n* (%)	Relative risk ratio (95% CI), *p*
No/don’t know	627 (88%)	47 (7%)	Ref	22 (3%)	Ref	18 (3%)	Ref
Yes	424 (88%)	28 (6%)	RRR = 0.88 (0.54–1.43), *p* = .61 aRRR = 0.91 (0.55–1.48), *p* = .69	18 (4%)	RRR = 1.21 (0.64–2.28), *p* = .56 aRRR = 1.18 (0.62–2.25), *p* = .62	14 (3%)	RRR = 1.15 (0.57–2.34), *p* = .70 aRRR = 1.10 (0.53–2.29), *p* = .79
3) Uptake of past 30-day smoking, vaping, or both among those who neither smoked nor vaped in the past 30 days at baseline (*n* = 2375):							
	No past 30-day smoking or vaping (*n* = 2,182; ref)	Initiation of past 30-day smoking only (*n* = 62)		Initiation of past 30-day vaping only (*n* = 88)		Initiation of both (*n* = 43)	
Perception that vaping is less harmful than smoking	*n* (%)	*n* (%)	Relative risk ratio (95% CI), p	*n* (%)	Relative risk ratio (95% CI), *p*	*n* (%)	Relative risk ratio (95% CI), *p*
No/don’t know	1247 (93%)	31 (2%)	Ref	46 (3%)	Ref	22 (2%)	Ref
Yes	935 (91%)	31 (3%)	RRR = 1.33 (0.80–2.21), *p* = .26 aRRR = 1.38 (0.83-2.31), *p* = .22	42 (4%)	RRR = 1.22 (0.79–1.87), *p* = .37 aRRR = 1.33 (0.86-2.04), *p* = .20	21 (2%)	RRR = 1.27 (0.70–2.33), *p* = .43 aRRR = 1.29 (0.70–2.40), *p* = .41

RRR = unadjusted relative risk ratio; aRRR = unadjusted relative risk ratio (adjusted for baseline questionnaire completed, sex assigned at birth, race/ethnicity, unemployed/not in education at age 22, mother/partner occupation, mother smoked during first 3 months of pregnancy); 95% CI = 95% Confidence Interval. The full models are shown in Supplementary Tables S1–S6.

#### Initiation of Ever Smoking and/or Vaping

Among those who had never vaped nor smoked at baseline (*n* = 1198), 6% (*n* = 75) had ever smoked only at follow-up, 3% (*n* = 40) had ever vaped only at follow-up, and 3% (*n* = 32) had ever tried both. There was little evidence for any associations between vaping harm perceptions and initiation of ever smoking, vaping, or both (Table 2).

#### Uptake of Past 30-day Smoking and/or Vaping

Among those who had neither vaped nor smoked in the past 30 days at baseline (*n* = 2375), 3% (*n* = 62) had smoked in the past 30 days at follow-up, 4% (*n* = 88) had vaped in the past 30 days at follow-up, and 2% (*n* = 43) had done both. There was little evidence for any associations between vaping harm perceptions and uptake of past 30-day smoking, vaping, or both (Table 2).

#### Sensitivity Analyses

In sensitivity analyses separating “don’t know” from “equally/more harmful than smoking” (Supplementary Table S7), among those who had smoked but did not vape in the past 30 days at baseline, the perception that vaping is less harmful than smoking was associated with increased likelihood of stopping smoking and now vaping compared with “don’t know” responses. There was no clear evidence of a difference between less and equally/more harmful than smoking. Similarly, among those who had never smoked nor vaped at baseline, the perception that vaping is less harmful than smoking vs. don’t know was associated with increased likelihood of initiating vaping only although sample sizes are too small for meaningful conclusions. Interpretation of other findings remained unchanged.

## Discussion

To the best of our knowledge, this is the first study among young adults in England to assess whether vaping harm perceptions are longitudinally associated with vaping and smoking behaviors. Among young adults who smoked at age 23/24, accurately perceiving vaping as less harmful than smoking was associated with stopping smoking and switching to vaping at age 30. Among young adults who did not smoke and/or vape at age 23/24, there was little evidence that harm perceptions predicted taking up smoking and/or vaping at age 30; however, this conclusion is limited by small numbers of young adults who started smoking and/or vaping during the study.

Results are consistent with prior research among adults finding that accurate vaping harm perceptions can predict quitting smoking as well as starting vaping.^1,3^ Demonstrating this association among *young adults* is important because the earlier someone stops smoking the better their health outcomes,^17^ and this group have more inaccurate vaping perceptions than older adults.^18^ Interventions to improve knowledge about vaping for tobacco harm reduction among young adults who smoke are needed, but none have been evaluated in the United Kingdom that specifically target this group.^1,3^

Results are inconsistent with prior research among nicotine-naïve young people finding that relative harm perceptions predict vaping initiation at the individual level.^1,3^ Most prior work here is from the United States, with follow-up shorter than six years and larger samples.^1,3^ The inconsistency in findings may be attributable to cultural, market, and/or regulatory differences between the United Kingdom and United States, low sample sizes in our study, and/or long follow-up during which associations between perceptions and behaviors may be less pronounced. However, our findings are consistent with observed discrepancies between young adults’ harm perceptions and the prevalence of vaping at the population level in England (ie, that perceptions are worsening while prevalence is increasing).^18,19^ Relative vaping harm perceptions may therefore not be as important for vaping initiation among nicotine-naïve young people as some evidence suggests. Cross-country studies with larger samples and more frequent assessments of vaping/smoking perceptions and behaviors are required.

This study has limitations. First, small sample sizes limit confidence in the findings for initiation of smoking and vaping. Second, smoking and vaping behaviors were not biologically verified and there is misreporting among the ALSPAC cohort.^20^ Third, findings may not generalize to recent years—baseline data were from 2015 to 2017, when vaping prevalence was lower,^19^ the e-cigarette market was very different (eg, before the widespread availability of disposables such as “Elf Bar”^21^), and perceptions were more accurate.^18^ Fourth, data were not nationally representative and there was an underrepresentation of males and those not in employment or education (among whom smoking is higher^22^) as well as racialized minorities. There was also substantial, inequitable, loss to follow-up, and complete case analyses were used. Findings may thus not generalize to young adults across England, particularly those who are more likely to smoke. Future research should aim to replicate these findings using larger, nationally representative samples and more recent data with biologically verified outcomes. Strengths include a six-year follow-up and consideration of both smoking and vaping outcomes, unlike previous studies which typically have shorter follow-up and mainly assess only vaping outcomes.^3^

In conclusion, among young adults who smoke, perceiving vaping as less harmful than smoking was associated with switching to vaping six years later. Few young adults who did not smoke or vape initiated either behavior during the study, and there was little evidence that this was predicted by harm perceptions.

## Supplementary Material

Supplementary material is available at *Nicotine and Tobacco Research* online.

ntaf018_suppl_Supplementary_Material

## Data Availability

Access to ALSPAC data is through a system of managed open access (http://www.bristol.ac.uk/alspac/researchers/access/).
