# Supplementary material for: Perceived Harm of Vaping Relative to Smoking and Associations With Subsequent Smoking and Vaping Behaviors Among Young Adults: Evidence From a UK Cohort Study
Source: Nicotine Tob Res. 2025 Feb 14;27(8):1479–85. doi: 10.1093/ntr/ntaf018 (PMC12280168; doi:10.1093/ntr/ntaf018)
Supplement: ntaf018_suppl_Supplementary_Material [file ntaf018_suppl_supplementary_material.docx]

**Figure S1. STROBE flow chart of sample recruitment and exclusion.**

**
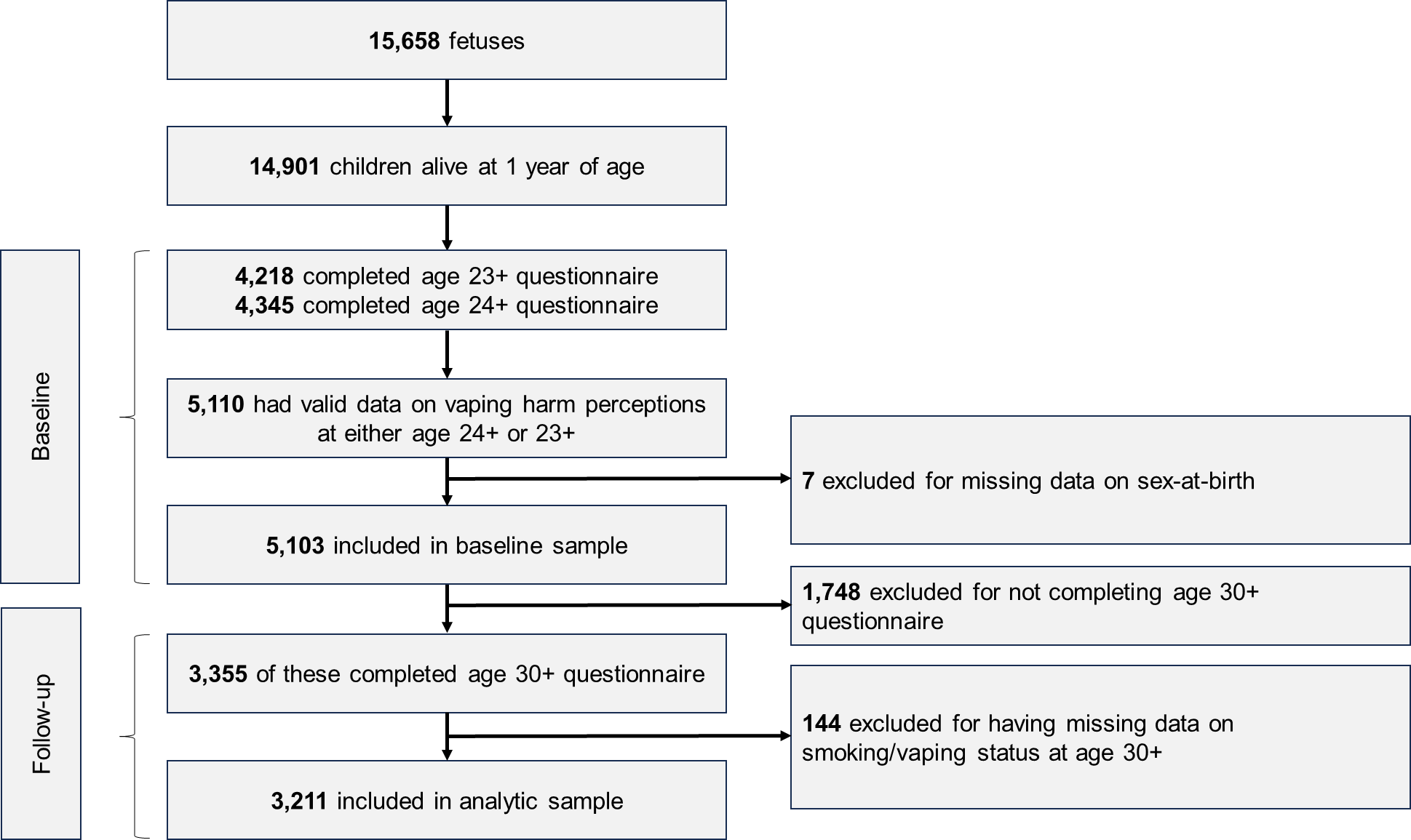
**

**Measures**

*Perceiving vaping as less harmful than smoking (exposure).* At all questionnaires (23+, 24+, 30+), respondents were asked “Compared to regular cigarettes, do you think electronic cigarettes/vaping devices are more harmful, less harmful or equally as harmful to health?” with response options (a) more harmful, (b) equally as harmful, (c) less harmful, (d) don’t know. For analysis, the exposure was dichotomised into accurate ‘less harmful’ (c) and all other, responses (a, b and d), consistent with prior work.^5,12^ As above, data were primarily from the age 24+ questionnaire but, where data were missing, data were imputed the 23+ questionnaire.

*Smoking and vaping behaviours (outcomes).* At all questionnaires (23+, 24+, 30+), respondents were asked “Have you ever smoked a whole cigarette (including roll-ups)?” (response options: Yes, No) and those who responded ‘yes’ were subsequently asked “Have you smoked any cigarettes in the past 30 days?” (response options: Yes, No). The vaping measures differed slightly at 23+/24+ and 30. In the 23+/24+ questionnaires, respondents were asked “Have you ever used/vaped an electronic cigarette (e-cigarette) or other vaping device? (response options: Yes, No) and those who responded ‘yes’ were subsequently asked “Do you currently use/vape electronic cigarettes or other vaping devices?” (response options: Yes, No). In the 30+ questionnaire, respondents were asked “Have you ever used/vaped an electronic cigarette or other vaping device (either nicotine-containing or nicotine-free devices)?” (response options: Yes, No) and those who responded ‘yes’ were subsequently asked “Have you used/vaped electronic cigarettes or other vaping devices in the past 30 days?” (response options: Yes, No). These measures were used to construct the following outcomes:

1. **Stopping smoking (including now vaping)**. Among those who only smoked (did not vape) in the past 30 days at baseline: (1-ref. category) past 30-day smoking only at 30+ (i.e., still smoking); (2) no past 30-day smoking/vaping at 30+ (i.e., stopped smoking, also not vaping); (3) past 30-day vaping only at 30+ (i.e., stopped smoking and now vaping); (4) past 30-day vaping and smoking at 30+ (i.e., still smoking and also now vaping). Categories 1-4 are mutually exclusive.
2. **Initiating ever smoking, vaping, or both.** Among those who had never vaped or smoked at baseline: (1-ref. category) never smoked/vaped at 30+ (i.e., abstained); (2) ever smoked only at 30+ (i.e., tried smoking); (3) ever vaping only at 30+ (i.e., tried vaping), (4) ever smoked and vaped at 30+ (i.e., tried both). Categories 1-4 are mutually exclusive.
3. **Uptake of past 30-day smoking, vaping, or both.** Among those who had not vaped or smoked in the past 30 days at baseline: (1-ref. category) no past 30-day smoking/vaping at 30+ (i.e., no uptake of current smoking/vaping); (2) past 30-day smoking only at 30+ (i.e., uptake of current smoking); (3) past 30-day vaping only at 30+ (i.e., uptake of current vaping); (4) past 30-day smoking and vaping at 30+ (i.e., uptake of both). Categories 1-4 are mutually exclusive.

*Confounders.* Potential confounders were: baseline questionnaire completed (23+, 24+); sex assigned at birth (male, female)^13^; race/ethnicity (white, racialised minorities, missing)^13^; unemployed or not in any form of education/training at age 22 (yes, no, missing)^13^; mother/partner occupation (derived from the lowest number available from either mother or partner: I-professional, II-managerial and technical, III-skilled manual or nonmanual, IV-semiskilled, V-unskilled, missing^14^; for this paper, IV and V were grouped due to low sample sizes for V-unskilled); mother smoking during the first 3 months of pregnancy (yes, no, missing).^13^ Covariates were selected and coded to be consistent with prior work assessing smoking and vaping using ALSPAC data.^13,14^ Due to large amounts of missing data on race/ethnicity, employment or education/training, mother/partner occupation, and mother smoking (Table 1), ‘missing’ was included as a unique category for these covariates to maximise sample size.

**Additional analyses and results: stability of the perceived harm of vaping relative to smoking over time**

To assess whether vaping harm perceptions were stable over time, we reported them at baseline and follow-up and compared them using a McNemar's χ2 test. We found that the prevalence of accurately perceiving vaping as less harmful than smoking in the overall sample was similar at baseline (age 23+/24+; 45%) compared to follow-up (age 30+; 46%) (p=.39). However, one third of respondents (n=1,044; 33%) changed their perception between baseline and follow-up, with around half (n=536) going from perceiving vaping as equally/more harmful than smoking, or don’t know, to less harmful, and the other half (n=508) going from perceiving vaping as less harmful than smoking to equally/more harmful or don’t know.

**Table S1. Among those who currently only smoked at baseline (age 23+/24+), unadjusted associations between** **perceived harm of vaping relative to smoking at baseline and subsequent past 30-day smoking/vaping at age 30+ (n=687).**

|  | Still smoking only (n=220; ref) |  | Stopped smoking, also not vaping (n=253) | | |  | | Stopped smoking and now vaping (n=93) | | | |  | | Still smoking and now vaping (n=121) | | | |  |
| --- | --- | --- | --- | --- | --- | --- | --- | --- | --- | --- | --- | --- | --- | --- | --- | --- | --- | --- |
|  | n (%) |  | n (%) | RRR (95% CI) | p | |  | | n (%) | RRR (95% CI) | p | |  | | n (%) | RRR (95% CI) | P | |
| **Perception that vaping is less harmful than smoking** |  |  |  |  |  | |  | |  |  |  | |  | |  |  |  | |
| No/don’t know | 139 (35%) |  | 139 (35%) | Ref |  | |  | | 50 (13%) | Ref |  | |  | | 68 (17%) | Ref |  | |
| Yes | 81 (28%) |  | 114 (40%) | 1.41 (0.97-2.04) | .07 | |  | | 43 (15%) | 1.48 (0.90-2.41) | .12 | |  | | 53 (18%) | 1.34 (0.85-2.10) | .21 | |
| **Baseline questionnaire completed** |  |  |  |  |  | |  | |  |  |  | |  | |  |  |  | |
| 23+ | 31 (31%) |  | 37 (37%) | Ref |  | |  | | 14 (14%) | Ref |  | |  | | 17 (17%) | Ref |  | |
| 24+ | 189 (32%) |  | 216 (37%) | 0.96 (0.57-1.60) | .87 | |  | | 79 (13%) | 0.93 (0.47-1.83) | .82 | |  | | 104 (18%) | 1.00 (0.53-1.90) | .99 | |
| **Sex assigned at birth** |  |  |  |  |  | |  | |  |  |  | |  | |  |  |  | |
| Female | 155 (32%) |  | 182 (37%) | Ref |  | |  | | 75 (15%) | Ref |  | |  | | 75 (15%) | Ref |  | |
| Male | 65 (33%) |  | 71 (36%) | 0.93 (0.62-1.39) | .72 | |  | | 18 (9%) | 0.57 (0.32-1.03) | .06 | |  | | 46 (23%) | 1.46 (0.92-2.33) | .11 | |
| **Race/ethnicity** |  |  |  |  |  | |  | |  |  |  | |  | |  |  |  | |
| White | 190 (33%) |  | 220 (38%) | Ref |  | |  | | 78 (13%) | Ref |  | |  | | 95 (16%) | Ref |  | |
| Racialised minorities | 5 (25%) |  | 7 (35%) | 1.21 (0.38-3.87) | .75 | |  | | <5^1^ (15%) | 1.46 (0.34-6.26) | .61 | |  | | 5 (25%) | 2.00 (0.57-7.08) | .28 | |
| Missing | 25 (30%) |  | 26 (31%) | 0.90 (0.50-1.61) | .72 | |  | | 12 (14%) | 1.17 (0.56-2.44) | .68 | |  | | 21 (25%) | 1.68 (0.89-3.16) | .11 | |
| **Unemployed/not in education, age 22** |  |  |  |  |  | |  | |  |  |  | |  | |  |  |  | |
| No | 149 (32%) |  | 176 (37%) | Ref |  | |  | | 65 (14%) | Ref |  | |  | | 83 (18%) | Ref |  | |
| Yes | 13 (45%) |  | 11 (38%) | 0.72 (0.31-1.65) | .43 | |  | | <5^1^ (7%) | 0.35 (0.08-1.61) | .18 | |  | | <5^1^ (10%) | 0.41 (0.11-1.50) | .18 | |
| Missing | 58 (32%) |  | 66 (36%) | 0.96 (0.64-1.46) | .86 | |  | | 26 (14%) | 1.03 (0.59-1.78) | .92 | |  | | 35 (19%) | 1.08 (0.66-1.78) | .75 | |
| **Mother/partner occupation** |  |  |  |  |  | |  | |  |  |  | |  | |  |  |  | |
| Professional | 30 (32%) |  | 39 (42%) | Ref |  | |  | | 9 (10%) | Ref |  | |  | | 15 (16%) | Ref |  | |
| Managerial and technical | 81 (32%) |  | 101 (40%) | 0.96 (0.55-1.68) | .88 | |  | | 28 (11%) | 1.15 (0.49-2.72) | .75 | |  | | 45 (18%) | 1.11 (0.54-2.28) | .77 | |
| Skilled manual or nonmanual | 52 (32%) |  | 55 (33%) | 0.81 (0.44-1.50) | .51 | |  | | 32 (19%) | 2.05 (0.86-4.87) | .10 | |  | | 26 (16%) | 1.00 (0.46-2.18) | 1.00 | |
| Semiskilled manual/unskilled | 5 (50%) |  | <5^1^ (40%) | 0.62 (0.15-2.49) | .50 | |  | | <5^1^ (10%) | 0.67 (0.07-6.47) | .73 | |  | | <5^1^ (<1%) | - |  | |
| Missing | 52 (32%) |  | 54 (33%) | 0.80 (0.43-1.47) | .47 | |  | | 23 (14%) | 1.47 (0.60-3.60) | .39 | |  | | 35 (21%) | 1.35 (0.63-2.86) | .44 | |
| **Mother smoked during the first 3 months of pregnancy** |  |  |  |  |  | |  | |  |  |  | |  | |  |  |  | |
| No | 152 (31%) |  | 201 (41%) | Ref |  | |  | | 61 (12%) | Ref |  | |  | | 76 (16%) | Ref |  | |
| Yes | 51 (39%) |  | 28 (21%) | 0.42 (0.25-0.69) | <.01 | |  | | 25 (19%) | 1.22 (0.7-2.15) | .49 | |  | | 28 (21%) | 1.10 (0.64-1.88) | .73 | |
| Missing | 17 (26%) |  | 24 (37%) | 1.07 (0.55-2.06) | .85 | |  | | 7 (11%) | 1.03 (0.41-2.6) | .96 | |  | | 17 (26%) | 2.00 (0.97-4.14) | .06 | |

RRR=unadjusted Relative Risk Ratio; 95% CI=95% Confidence Interval.

^1^ Cell counts less than 5 (including zero) are reported as <5 in line with ALSPAC’s requirements <https://www.bristol.ac.uk/media-library/sites/alspac/documents/alspac-publications-checklist.pdf>.

**Table S2. Among those who currently only smoked at baseline (age 23+/24+), adjusted associations between** **perceived harm of vaping relative to smoking at baseline and subsequent past 30-day smoking/vaping at age 30+ (n=687).**

|  | Still smoking only (n=220; ref) |  | Stopped smoking, also not vaping (n=253) | | |  | | Stopped smoking and now vaping (n=93) | | | |  | | Still smoking and now vaping (n=121) | | | |  |
| --- | --- | --- | --- | --- | --- | --- | --- | --- | --- | --- | --- | --- | --- | --- | --- | --- | --- | --- |
|  | n (%) |  | n (%) | aRRR (95% CI) | p | |  | | n (%) | aRRR (95% CI) | p | |  | | n (%) | aRRR (95% CI) | p | |
| **Perception that vaping is less harmful than smoking** |  |  |  |  |  | |  | |  |  |  | |  | |  |  |  | |
| No/don’t know | 139 (35%) |  | 139 (35%) | Ref |  | |  | | 50 (13%) | Ref |  | |  | | 68 (17%) | Ref |  | |
| Yes | 81 (28%) |  | 114 (40%) | 1.37 (0.94-2.01) | .10 | |  | | 43 (15%) | 1.69 (1.02-2.81) | .04 | |  | | 53 (18%) | 1.31 (0.82-2.09) | .26 | |
| **Baseline questionnaire completed** |  |  |  |  |  | |  | |  |  |  | |  | |  |  |  | |
| 23+ | 31 (31%) |  | 37 (37%) | Ref |  | |  | | 14 (14%) | Ref |  | |  | | 17 (17%) | Ref |  | |
| 24+ | 189 (32%) |  | 216 (37%) | 0.91 (0.54-1.55) | .74 | |  | | 79 (13%) | 0.90 (0.45-1.82) | .77 | |  | | 104 (18%) | 1.13 (0.59-2.18) | .71 | |
| **Sex assigned at birth** |  |  |  |  |  | |  | |  |  |  | |  | |  |  |  | |
| Female | 155 (32%) |  | 182 (37%) | Ref |  | |  | | 75 (15%) | Ref |  | |  | | 75 (15%) | Ref |  | |
| Male | 65 (33%) |  | 71 (36%) | 0.91 (0.60-1.38) | .67 | |  | | 18 (9%) | 0.57 (0.31-1.04) | .07 | |  | | 46 (23%) | 1.52 (0.94-2.46) | .09 | |
| **Race/ethnicity** |  |  |  |  |  | |  | |  |  |  | |  | |  |  |  | |
| White | 190 (33%) |  | 220 (38%) | Ref |  | |  | | 78 (13%) | Ref |  | |  | | 95 (16%) | Ref |  | |
| Racialised minorities | 5 (25%) |  | 7 (35%) | 1.21 (0.37-3.96) | .76 | |  | | <5^1^ (15%) | 1.74 (0.39-7.70) | .47 | |  | | 5 (25%) | 2.01 (0.55-7.29) | .29 | |
| Missing | 25 (30%) |  | 26 (31%) | 0.61 (0.21-1.75) | .36 | |  | | 12 (14%) | 1.48 (0.47-4.70) | .50 | |  | | 21 (25%) | 1.21 (0.39-3.74) | .75 | |
| **Unemployed/not in education, age 22** |  |  |  |  |  | |  | |  |  |  | |  | |  |  |  | |
| No | 149 (32%) |  | 176 (37%) | Ref |  | |  | | 65 (14%) | Ref |  | |  | | 83 (18%) | Ref |  | |
| Yes | 13 (45%) |  | 11 (38%) | 0.71 (0.30-1.65) | .43 | |  | | <5^1^ (7%) | 0.38 (0.08-1.76) | .22 | |  | | <5^1^ (10%) | 0.38 (0.11-1.41) | .15 | |
| Missing | 58 (32%) |  | 66 (36%) | 1.03 (0.67-1.58) | .90 | |  | | 26 (14%) | 1.07 (0.61-1.88) | .81 | |  | | 35 (19%) | 1.06 (0.64-1.77) | .82 | |
| **Mother/partner occupation** |  |  |  |  |  | |  | |  |  |  | |  | |  |  |  | |
| Professional | 30 (32%) |  | 39 (42%) | Ref |  | |  | | 9 (10%) | Ref |  | |  | | 15 (16%) | Ref |  | |
| Managerial and technical | 81 (32%) |  | 101 (40%) | 1.03 (0.58-1.82) | .92 | |  | | 28 (11%) | 1.13 (0.47-2.72) | .78 | |  | | 45 (18%) | 1.19 (0.57-2.49) | .64 | |
| Skilled manual or nonmanual | 52 (32%) |  | 55 (33%) | 0.99 (0.52-1.86) | .96 | |  | | 32 (19%) | 2.09 (0.85-5.18) | .11 | |  | | 26 (16%) | 1.11 (0.49-2.52) | .81 | |
| Semiskilled manual/unskilled | 5 (50%) |  | <5^1^ (40%) | 0.83 (0.19-3.56) | .80 | |  | | <5^1^ (10%) | 0.68 (0.07-6.86) | .74 | |  | | <5^1^ (<1%) | - | .99 | |
| Missing | 52 (32%) |  | 54 (33%) | 0.94 (0.46-1.92) | .87 | |  | | 23 (14%) | 1.36 (0.50-3.75) | .55 | |  | | 35 (21%) | 1.08 (0.44-2.65) | .87 | |
| **Mother smoked during the first 3 months of pregnancy** |  |  |  |  |  | |  | |  |  |  | |  | |  |  |  | |
| No | 152 (31%) |  | 201 (41%) | Ref |  | |  | | 61 (12%) | Ref |  | |  | | 76 (16%) | Ref |  | |
| Yes | 51 (39%) |  | 28 (21%) | 0.44 (0.26-0.74) | <.01 | |  | | 25 (19%) | 1.13 (0.62-2.03) | .70 | |  | | 28 (21%) | 1.13 (0.64-1.98) | .68 | |
| Missing | 17 (26%) |  | 24 (37%) | 1.77 (0.59-5.29) | .31 | |  | | 7 (11%) | 0.71 (0.19-2.68) | .61 | |  | | 17 (26%) | 1.96 (0.60-6.42) | .27 | |

aRRR=adjusted Relative Risk Ratio; 95% CI=95% Confidence Interval.

^1^ Cell counts less than 5 (including zero) are reported as <5 in line with ALSPAC’s requirements <https://www.bristol.ac.uk/media-library/sites/alspac/documents/alspac-publications-checklist.pdf>.

**Table S3. Among those who had never vaped nor smoked at baseline (age 23+/24+), unadjusted associations between** **perceived harm of vaping relative to smoking at baseline and subsequent ever vaping/smoking at age 30+ (n=1,198).**

|  | No initiation (n=1,051; ref) |  | Initiation of ever smoking only (n=75) | | |  | | Initiation of ever vaping only (n=40) | | |  | | Initiation of both (n=32) | | |
| --- | --- | --- | --- | --- | --- | --- | --- | --- | --- | --- | --- | --- | --- | --- | --- |
|  | n (%) |  | n (%) | RRR (95% CI) | p | |  | n (%) | RRR (95% CI) | p |  | n (%) | | RRR (95% CI) | p |
| **Perception that vaping is less harmful than smoking** |  |  |  |  |  | |  |  |  |  |  |  | |  |  |
| No/don’t know | 627 (88%) |  | 47 (7%) | Ref |  | |  | 22 (3%) | Ref |  |  | 18 (3%) | | Ref |  |
| Yes | 424 (88%) |  | 28 (6%) | 0.88 (0.54-1.43) | .61 | |  | 18 (4%) | 1.21 (0.64-2.28) | .56 |  | 14 (3%) | | 1.15 (0.57-2.34) | .70 |
| **Baseline questionnaire completed** |  |  |  |  |  | |  |  |  |  |  |  | |  |  |
| 23+ | 109 (85%) |  | 13 (10%) | Ref |  | |  | <5^1^ (3%) | Ref |  |  | <5^1^ (2%) | | Ref |  |
| 24+ | 942 (88%) |  | 62 (6%) | 0.55 (0.29-1.04) | .06 | |  | 36 (3%) | 1.04 (0.36-2.98) | .94 |  | 30 (3%) | | 1.74 (0.41-7.36) | .46 |
| **Sex assigned at birth** |  |  |  |  |  | |  |  |  |  |  |  | |  |  |
| Female | 707 (88%) |  | 49 (6%) | Ref |  | |  | 27 (3%) | Ref |  |  | 21 (3%) | | Ref |  |
| Male | 344 (87%) |  | 26 (7%) | 1.09 (0.67-1.78) | .73 | |  | 13 (3%) | 0.99 (0.5-1.94) | .98 |  | 11 (3%) | | 1.08 (0.51-2.26) | .85 |
| **Race/ethnicity** |  |  |  |  |  | |  |  |  |  |  |  | |  |  |
| White | 902 (87%) |  | 71 (7%) | Ref |  | |  | 32 (3%) | Ref |  |  | 28 (3%) | | Ref |  |
| Racialised minorities | 41 (95%) |  | <5^1^ (<1%) | - |  | |  | <5^1^ (5%) | 1.38 (0.32-5.94) | .70 |  | <5^1^ (<1%) | | - |  |
| Missing | 108 (89%) |  | <5^1^ (3%) | 0.47 (0.17-1.31) | .15 | |  | 6 (5%) | 1.57 (0.64-3.83) | .33 |  | <5^1^ (3%) | | 1.19 (0.41-3.47) | .75 |
| **Unemployed/not in education, age 22** |  |  |  |  |  | |  |  |  |  |  |  | |  |  |
| No | 819 (89%) |  | 48 (5%) | Ref |  | |  | 28 (3%) | Ref |  |  | 26 (3%) | | Ref |  |
| Yes | 56 (88%) |  | 7 (11%) | 2.13 (0.92-4.93) | .08 | |  | <5^1^ (2%) | 0.52 (0.07-3.91) | .53 |  | <5^1^ (<1%) | | - |  |
| Missing | 176 (83%) |  | 20 (9%) | 1.94 (1.12-3.35) | .02 | |  | 11 (5%) | 1.83 (0.89-3.74) | .10 |  | 6 (3%) | | 1.07 (0.44-2.65) | .88 |
| **Mother/partner occupation** |  |  |  |  |  | |  |  |  |  |  |  | |  |  |
| Professional | 199 (87%) |  | 18 (8%) | Ref |  | |  | 7 (3%) | Ref |  |  | 4 (2%) | | Ref |  |
| Managerial and technical | 363 (86%) |  | 25 (6%) | 0.76 (0.41-1.43) | .40 | |  | 19 (5%) | 1.49 (0.61-3.60) | .38 |  | 14 (3%) | | 1.92 (0.62-5.91) | .26 |
| Skilled manual or nonmanual | 271 (88%) |  | 22 (7%) | 0.90 (0.47-1.72) | .74 | |  | 5 (2%) | 0.52 (0.16-1.68) | .28 |  | 9 (3%) | | 1.65 (0.50-5.44) | .41 |
| Semiskilled manual/unskilled | 16 (89%) |  | <5^1^ (6%) | 0.69 (0.09-5.51) | .73 | |  | 1 (6%) | 1.78 (0.21-15.35) | .60 |  | <5^1^ (<1%) | | - |  |
| Missing | 202 (90%) |  | 9 (4%) | 0.49 (0.22-1.12) | .09 | |  | 8 (4%) | 1.13 (0.40-3.16) | .82 |  | 5 (2%) | | 1.23 (0.33-4.65) | .76 |
| **Mother smoked during the first 3 months of pregnancy** |  |  |  |  |  | |  |  |  |  |  |  | |  |  |
| No | 862 (88%) |  | 64 (7%) | Ref |  | |  | 31 (3%) | Ref |  |  | 23 (2%) | | Ref |  |
| Yes | 108 (86%) |  | 8 (6%) | 1.00 (0.47-2.14) | 1.00 | |  | <5^1^ (3%) | 1.03 (0.36-2.97) | .96 |  | 6 (5%) | | 2.08 (0.83-5.23) | .12 |
| Missing | 81 (88%) |  | <5^1^ (3%) | 0.50 (0.15-1.62) | .25 | |  | 5 (5%) | 1.72 (0.65-4.54) | .28 |  | <5^1^ (3%) | | 1.39 (0.41-4.72) | .60 |

RRR=unadjusted Relative Risk Ratio; 95% CI=95% Confidence Interval.

^1^ Cell counts less than 5 (including zero) are reported as <5 in line with ALSPAC’s requirements <https://www.bristol.ac.uk/media-library/sites/alspac/documents/alspac-publications-checklist.pdf>.

**Table S4. Among those who had never vaped nor smoked at baseline (age 23+/24+), adjusted associations between** **perceived harm of vaping relative to smoking at baseline and subsequent ever smoking/vaping at age 30+ (n=1,198).**

|  | No initiation (n=1,051; ref) |  | Initiation of ever smoking only (n=75) | | |  | | Initiation of ever vaping only (n=40) | | |  | | Initiation of both (n=32) | | | |
| --- | --- | --- | --- | --- | --- | --- | --- | --- | --- | --- | --- | --- | --- | --- | --- | --- |
|  | n (%) |  | n (%) | aRRR (95% CI) | p |  | n (%) | | aRRR (95% CI) | p |  | n (%) | | aRRR (95% CI) | p |  |
| **Perception that vaping is less harmful than smoking** |  |  |  |  |  |  |  | |  |  |  |  | |  |  |  |
| No/don’t know | 627 (88%) |  | 47 (7%) | Ref |  |  | 22 (3%) | | Ref |  |  | 18 (3%) | | Ref |  |  |
| Yes | 424 (88%) |  | 28 (6%) | 0.91 (0.55-1.48) | .69 |  | 18 (4%) | | 1.18 (0.62-2.25) | .62 |  | 14 (3%) | | 1.10 (0.53-2.29) | .79 |  |
| **Baseline questionnaire completed** |  |  |  |  |  |  |  | |  |  |  |  | |  |  |  |
| 23+ | 109 (85%) |  | 13 (10%) | Ref |  |  | <5^1^ (3%) | | Ref |  |  | <5^1^ (2%) | | Ref |  |  |
| 24+ | 942 (88%) |  | 62 (6%) | 0.65 (0.34-1.26) | .20 |  | 36 (3%) | | 1.17 (0.4-3.47) | .77 |  | 30 (3%) | | 1.88 (0.43-8.18) | .40 |  |
| **Sex assigned at birth** |  |  |  |  |  |  |  | |  |  |  |  | |  |  |  |
| Female | 344 (87%) |  | 26 (7%) | Ref |  |  | 27 (3%) | | Ref |  |  | 21 (3%) | | Ref |  |  |
| Male | 707 (88%) |  | 49 (6%) | 0.97 (0.58-1.60) | .90 |  | 13 (3%) | | 0.96 (0.48-1.9) | .90 |  | 11 (3%) | | 1.09 (0.51-2.31) | .83 |  |
| **Race/ethnicity** |  |  |  |  |  |  |  | |  |  |  |  | |  |  |  |
| White | 902 (87%) |  | 71 (7%) | Ref |  |  | 32 (3%) | | Ref |  |  | 28 (3%) | | Ref |  |  |
| Racialised minorities | 41 (95%) |  | <5^1^ (<1%) | - |  |  | <5^1^ (5%) | | 1.43 (0.32-6.36) | .64 |  | <5^1^ (<1%) | | - | .99 |  |
| Missing | 108 (89%) |  | <5^1^ (3%) | 0.61 (0.12-3.07) | .55 |  | 6 (5%) | | 1.59 (0.27-9.26) | .61 |  | <5^1^ (3%) | | 1.57 (0.21-11.65) | .66 |  |
| **Unemployed/not in education, age 22** |  |  |  |  |  |  |  | |  |  |  |  | |  |  |  |
| No | 819 (89%) |  | 48 (5%) | Ref |  |  | 28 (3%) | | Ref |  |  | 26 (3%) | | Ref |  |  |
| Yes | 56 (88%) |  | 7 (11%) | 2.16 (0.92-5.06) | .08 |  | <5^1^ (2%) | | 0.51 (0.07-3.85) | .51 |  | <5^1^ (<1%) | | - | .99 |  |
| Missing | 176 (83%) |  | 20 (9%) | 1.90 (1.07-3.39) | .03 |  | 11 (5%) | | 1.88 (0.89-3.96) | .10 |  | 6 (3%) | | 1.12 (0.44-2.85) | .82 |  |
| **Mother/partner occupation** |  |  |  |  |  |  |  | |  |  |  |  | |  |  |  |
| Professional | 199 (87%) |  | 18 (8%) | Ref |  |  | 7 (3%) | | Ref |  |  | <5^1^ (2%) | | Ref |  |  |
| Managerial and technical | 363 (86%) |  | 25 (6%) | 0.76 (0.40-1.44) | .40 |  | 19 (5%) | | 1.43 (0.59-3.49) | .43 |  | 14 (3%) | | 1.76 (0.57-5.48) | .33 |  |
| Skilled manual or nonmanual | 271 (88%) |  | 22 (7%) | 0.90 (0.46-1.75) | .76 |  | 5 (2%) | | 0.50 (0.15-1.61) | .25 |  | 9 (3%) | | 1.47 (0.44-4.95) | .53 |  |
| Semiskilled manual/unskilled | 16 (89%) |  | <5^1^ (6%) | 0.70 (0.08-5.84) | .74 |  | <5^1^ (6%) | | 1.59 (0.17-14.55) | .68 |  | <5^1^ (<1%) | | - | - |  |
| Missing | 202 (90%) |  | 9 (4%) | 0.63 (0.23-1.71) | .37 |  | 8 (4%) | | 0.58 (0.14-2.45) | .46 |  | 5 (2%) | | 0.63 (0.11-3.73) | .61 |  |
| **Mother smoked during the first 3 months of pregnancy** |  |  |  |  |  |  |  | |  |  |  |  | |  |  |  |
| No | 862 (88%) |  | 64 (7%) | Ref |  |  | 31 (3%) | | Ref |  |  | 23 (2%) | | Ref |  |  |
| Yes | 108 (86%) |  | 8 (6%) | 1.00 (0.46-2.21) | .99 |  | <5^1^ (3%) | | 1.01 (0.34-3.06) | .98 |  | 6 (5%) | | 2.19 (0.84-5.69) | .11 |  |
| Missing | 81 (88%) |  | <5^1^ (3%) | 0.92 (0.16-5.11) | .92 |  | 5 (5%) | | 1.70 (0.31-9.38) | .54 |  | <5^1^ (3%) | | 1.76 (0.22-14.07) | .59 |  |

aRRR=adjusted Relative Risk Ratio; 95% CI=95% Confidence Interval.

^1^ Cell counts less than 5 (including zero) are reported as <5 in line with ALSPAC’s requirements <https://www.bristol.ac.uk/media-library/sites/alspac/documents/alspac-publications-checklist.pdf>

**Table S5. Among those who had not vaped nor smoked in the past 30 days at baseline (age 23+/24+), unadjusted associations between** **perceived harm of vaping relative to smoking at baseline and subsequent past 30-day smoking/vaping at age 30+ (n=2,375).**

|  | No past 30-day smoking or vaping (n=2,182; ref) |  | Initiation of past 30-day smoking only (n=62) | | |  | Initiation of past 30-day vaping only (n=88) | | | |  | Initiation of both (n=43) | | | | |
| --- | --- | --- | --- | --- | --- | --- | --- | --- | --- | --- | --- | --- | --- | --- | --- | --- |
|  | n (%) |  | n (%) | RRR (95% CI) | p |  | n (%) | RRR (95% CI) | p |  | | | n (%) | RRR (95% CI) | p |  |
| **Perception that vaping is less harmful than smoking** |  |  |  |  |  |  |  |  |  |  | | |  |  |  |  |
| No/don’t know | 1247 (93%) |  | 31 (2%) | Ref |  |  | 46 (3%) | Ref |  |  | | | 22 (2%) | Ref |  |  |
| Yes | 935 (91%) |  | 31 (3%) | 1.33 (0.80-2.21) | .26 |  | 42 (4%) | 1.22 (0.79-1.87) | .37 |  | | | 21 (2%) | 1.27 (0.70-2.33) | .43 |  |
| **Baseline questionnaire completed** |  |  |  |  |  |  |  |  |  |  | | |  |  |  |  |
| 23+ | 212 (88%) |  | 8 (3%) | Ref |  |  | 10 (4%) | Ref |  |  | | | 10 (4%) | Ref |  |  |
| 24+ | 1,970 (92%) |  | 54 (3%) | 0.73 (0.34-1.55) | .41 |  | 78 (4%) | 0.84 (0.43-1.65) | .61 |  | | | 33 (2%) | 0.36 (0.17-0.73) | .01 |  |
| **Sex assigned at birth** |  |  |  |  |  |  |  |  |  |  | | |  |  |  |  |
| Female | 1490 (92%) |  | 41 (3%) | Ref |  |  | 65 (4%) | Ref |  |  | | | 26 (2%) | Ref |  |  |
| Male | 692 (92%) |  | 21 (3%) | 1.10 (0.65-1.88) | .72 |  | 23 (3%) | 0.76 (0.47-1.24) | .27 |  | | | 17 (2%) | 1.41 (0.76-2.61) | .28 |  |
| **Race/ethnicity** |  |  |  |  |  |  |  |  |  |  | | |  |  |  |  |
| White | 1891 (92%) |  | 54 (3%) | Ref |  |  | 79 (4%) | Ref |  |  | | | 36 (2%) | Ref |  |  |
| Racialised minorities | 71 (91%) |  | <5^1^ (3%) | 0.99 (0.24-4.13) | .99 |  | <5^1^ (1%) | 0.34 (0.05-2.46) | .28 |  | | | <5^1^ (5%) | 2.96 (1.03-8.54) | .05 |  |
| Missing | 220 (93%) |  | 6 (3%) | 0.96 (0.41-2.25) | .92 |  | 8 (3%) | 0.87 (0.42-1.83) | .71 |  | | | <5^1^ (1%) | 0.72 (0.22-2.35) | .58 |  |
| **Unemployed/not in education, age 22** |  |  |  |  |  |  |  |  |  |  | | |  |  |  |  |
| No | 1644 (93%) |  | 40 (2%) | Ref |  |  | 58 (3%) | Ref |  |  | | | 30 (2%) | Ref |  |  |
| Yes | 104 (91%) |  | <5^1^ (1%) | 0.40 (0.05-2.90) | .36 |  | 5 (4%) | 1.36 (0.54-3.47) | .52 |  | | | <5^1^ (4%) | 2.11 (0.73-6.10) | .17 |  |
| Missing | 434 (89%) |  | 21 (4%) | 1.99 (1.16-3.41) | .01 |  | 25 (5%) | 1.63 (1.01-2.64) | .05 |  | | | 9 (2%) | 1.14 (0.54-2.41) | .74 |  |
| **Mother/partner occupation** |  |  |  |  |  |  |  |  |  |  | | |  |  |  |  |
| Professional | 375 (93%) |  | 8 (2%) | Ref |  |  | 9 (2%) | Ref |  |  | | | 10 (2%) | Ref |  |  |
| Managerial and technical | 801 (91%) |  | 31 (4%) | 1.81 (0.83-3.98) | .14 |  | 29 (3%) | 1.51 (0.71-3.22) | .29 |  | | | 18 (2%) | 0.84 (0.39-1.84) | .67 |  |
| Skilled manual or nonmanual | 544 (92%) |  | 14 (2%) | 1.21 (0.50-2.90) | .68 |  | 27 (5%) | 2.07 (0.96-4.45) | .06 |  | | | 7 (1%) | 0.48 (0.18-1.28) | .14 |  |
| Semiskilled manual/unskilled | 37 (88%) |  | <5^1^ (<1%) | - |  |  | <5^1^ (10%) | 4.50 (1.32-15.34) | .02 |  | | | <5^1^ (2%) | 1.01 (0.13-8.14) | .99 |  |
| Missing | 425 (92%) |  | 9 (2%) | 0.99 (0.38-2.60) | .99 |  | 19 (4%) | 1.86 (0.83-4.17) | .13 |  | | | 7 (2%) | 0.62 (0.23-1.64) | .33 |  |
| **Mother smoked during the first 3 months of pregnancy** |  |  |  |  |  |  |  |  |  |  | | |  |  |  |  |
| No | 1749 (92%) |  | 51 (3%) | Ref |  |  | 64 (3%) | Ref |  |  | | | 35 (2%) | Ref |  |  |
| Yes | 260 (90%) |  | 6 (2%) | 0.79 (0.34-1.86) | .59 |  | 16 (6%) | 1.68 (0.96-2.95) | .07 |  | | | 7 (2%) | 1.35 (0.59-3.06) | .48 |  |
| Missing | 173 (93%) |  | 5 (3%) | 0.99 (0.39-2.52) | .99 |  | 8 (4%) | 1.26 (0.60-2.68) | .54 |  | | | <5^1^ (1%) | 0.29 (0.04-2.12) | .22 |  |

RRR=unadjusted Relative Risk Ratio; 95% CI=95% Confidence Interval.

^1^ Cell counts less than 5 (including zero) are reported as <5 in line with ALSPAC’s requirements <https://www.bristol.ac.uk/media-library/sites/alspac/documents/alspac-publications-checklist.pdf>.

**Table S6. Among those who neither currently smoked nor currently vaped at baseline (age 23+/24+), adjusted associations between** **perceived harm of vaping relative to smoking at baseline and subsequent past 30-day smoking/vaping at age 30+ (n=2,375).**

|  | No past 30-day smoking or vaping (n=2,182; ref) |  | Initiation of past 30-day smoking only (n=62) | | |  | Initiation of past 30-day vaping only (n=88) | | |  | Initiation of both (n=43) | | | |
| --- | --- | --- | --- | --- | --- | --- | --- | --- | --- | --- | --- | --- | --- | --- |
|  | n (%) |  | n (%) | aRRR (95% CI) | p |  | n (%) | aRRR (95% CI) | p |  | | n (%) | aRRR (95% CI) | p |
| **Perception that vaping is less harmful than smoking** |  |  |  |  |  |  |  |  |  |  | |  |  |  |
| No/don’t know | 1247 (93%) |  | 31 (2%) | Ref |  |  | 46 (3%) | Ref |  |  | | 22 (2%) | Ref |  |
| Yes | 935 (91%) |  | 31 (3%) | 1.38 (0.83-2.31) | .22 |  | 42 (4%) | 1.33 (0.86-2.04) | .20 |  | | 21 (2%) | 1.29 (0.70-2.40) | .41 |
| **Baseline questionnaire completed** |  |  |  |  |  |  |  |  |  |  | |  |  |  |
| 23+ | 212 (88%) |  | 8 (3%) | Ref |  |  | 10 (4%) | Ref |  |  | | 10 (4%) | Ref |  |
| 24+ | 1,970 (92%) |  | 54 (3%) | 0.86 (0.70-0.39) | .70 |  | 78 (4%) | 0.90 (0.76-0.45) | .76 |  | | 33 (2%) | 0.36 (0.01-0.17) | .01 |
| **Sex assigned at birth** |  |  |  |  |  |  |  |  |  |  | |  |  |  |
| Female | 1490 (92%) |  | 41 (3%) | Ref |  |  | 65 (4%) | Ref |  |  | | 26 (2%) | Ref |  |
| Male | 692 (92%) |  | 21 (3%) | 1.01 (0.97-0.59) | .97 |  | 23 (3%) | 0.74 (0.24-0.45) | .24 |  | | 17 (2%) | 1.23 (0.53-0.65) | .53 |
| **Race/ethnicity** |  |  |  |  |  |  |  |  |  |  | |  |  |  |
| White | 1891 (92%) |  | 54 (3%) | Ref |  |  | 79 (4%) | Ref |  |  | | 36 (2%) | Ref |  |
| Racialised minorities | 71 (91%) |  | <5^1^ (3%) | 1.10 (0.90-0.26) | .90 |  | <5^1^ (1%) | 0.29 (0.23-0.04) | .23 |  | | <5^1^ (5%) | 2.84 (0.06-0.96) | .06 |
| Missing | 220 (93%) |  | 6 (3%) | 1.24 (0.79-0.25) | .79 |  | 8 (3%) | 0.40 (0.16-0.11) | .16 |  | | <5^1^ (1%) | 1.64 (0.54-0.33) | .54 |
| **Unemployed/not in education, age 22** |  |  |  |  |  |  |  |  |  |  | |  |  |  |
| No | 1644 (93%) |  | 40 (2%) | Ref |  |  | 58 (3%) | Ref |  |  | | 30 (2%) | Ref |  |
| Yes | 104 (91%) |  | <5^1^ (1%) | 0.39 (0.36-0.05) | .36 |  | 5 (4%) | 1.58 (0.34-0.61) | .34 |  | | <5^1^ (4%) | 1.80 (0.29-0.61) | .29 |
| Missing | 434 (89%) |  | 21 (4%) | 2.01 (0.01-1.15) | .01 |  | 25 (5%) | 1.60 (0.06-0.97) | .06 |  | | 9 (2%) | 0.95 (0.89-0.44) | .89 |
| **Mother/partner occupation** |  |  |  |  |  |  |  |  |  |  | |  |  |  |
| Professional | 375 (93%) |  | 8 (2%) | Ref |  |  | 9 (2%) | Ref |  |  | | 10 (2%) | Ref |  |
| Managerial and technical | 801 (91%) |  | 31 (4%) | 1.83 (0.14-0.83) | .14 |  | 29 (3%) | 1.47 (0.32-0.69) | .32 |  | | 18 (2%) | 0.85 (0.68-0.38) | .68 |
| Skilled manual or nonmanual | 544 (92%) |  | 14 (2%) | 1.23 (0.65-0.51) | .65 |  | 27 (5%) | 1.99 (0.08-0.92) | .08 |  | | 7 (1%) | 0.47 (0.14-0.17) | .14 |
| Semiskilled manual/unskilled | 37 (88%) |  | <5^1^ (<1%) | - | .99 |  | <5^1^ (10%) | 3.77 (0.04-1.07) | .04 |  | | <5^1^ (2%) | 0.91 (0.93-0.11) | .93 |
| Missing | 425 (92%) |  | 9 (2%) | 0.72 (0.60-0.21) | .60 |  | 19 (4%) | 1.97 (0.14-0.79) | .14 |  | | 7 (2%) | 0.72 (0.57-0.23) | .57 |
| **Mother smoked during the first 3 months of pregnancy** |  |  |  |  |  |  |  |  |  |  | |  |  |  |
| No | 1749 (92%) |  | 51 (3%) | Ref |  |  | 64 (3%) | Ref |  |  | | 35 (2%) | Ref |  |
| Yes | 260 (90%) |  | 6 (2%) | 0.79 (0.59-0.33) | .59 |  | 16 (6%) | 1.45 (0.21-0.81) | .21 |  | | 7 (2%) | 1.45 (0.39-0.62) | .39 |
| Missing | 173 (93%) |  | 5 (3%) | 1.40 (0.69-0.27) | .69 |  | 8 (4%) | 2.05 (0.26-0.58) | .26 |  | | <5^1^ (1%) | 0.23 (0.22-0.02) | .22 |

aRRR=adjusted Relative Risk Ratio; 95% CI=95% Confidence Interval.

^1^ Cell counts less than 5 (including zero) are reported as <5 in line with ALSPAC’s requirements <https://www.bristol.ac.uk/media-library/sites/alspac/documents/alspac-publications-checklist.pdf>.

**Table S7. Sensitivity analyses separating out ‘don’t know’ as a unique category when assessing associations between** **perceived harm of vaping relative to smoking at baseline and subsequent smoking and vaping at age 30+.**

|  |  |  |  |  |  |  |  |  | |  |  |
| --- | --- | --- | --- | --- | --- | --- | --- | --- | --- | --- | --- |
| 1. **Stopping smoking (including now vaping) among those who had only smoked in the past 30 days at baseline (n=687):** | | | | | | | | | | | |
|  | Still smoking only (n=220; ref) |  | Stopped smoking, also not vaping (n=253) | |  | Stopped smoking and now vaping (n=93) | |  | | Still smoking and now vaping (n=121) | |
| **Perception that vaping is less harmful than smoking** | n (%) |  | n (%) | aRRR (95% CI), p |  | n (%) | aRRR (95% CI), p |  | | n (%) | aRRR (95% CI), p |
| Yes | 81 (28%) |  | 114 (39%) | Ref |  | 43 (15%) | Ref |  | | 53 (18%) | Ref |
| No | 77 (35%) |  | 75 (34%) | 0.70 (0.45-1.09), p=.12 |  | 32 (15%) | 0.69 (0.39-1.22), p=.20 |  | | 37 (17%) | 0.76 (0.44-1.31), p=.34 |
| Don’t know | 62 (35%) |  | 64 (37%) | 0.76 (0.48-1.21), p=.25 |  | 18 (10%) | 0.48 (0.25-0.92), p=.03 |  | | 31 (18%) | 0.76 (0.43-1.34), p=.34 |
|  |  |  |  |  |  |  |  |  | |  |  |
| 1. **Initiating ever smoking, vaping, or both among those who had never smoked nor vaped at baseline (n=1,198):** | | | | | | | | | | | |
|  | No initiation (n=1,051; ref) |  | Initiation of ever smoking only (n=75) | |  | Initiation of ever vaping only (n=40) | |  | | Initiation of both (n=32) | |
| **Perception that vaping is less harmful than smoking** | n (%) |  | n (%) | aRRR (95% CI), p |  | n (%) | aRRR (95% CI), p |  | | n (%) | aRRR (95% CI), p |
| Yes | 424 (87%) |  | 28 (6%) | Ref |  | 18 (4%) | Ref |  | | 14 (3%) | Ref |
| No | 408 (87%) |  | 26 (6%) | 0.97 (0.55-1.70), p=.90 |  | 20 (4%) | 1.23 (0.63-2.39), p=.54 |  | | 13 (3%) | 0.99 (0.45-2.18), p=.98 |
| Don’t know | 219 (88%) |  | 21 (9%) | 1.32 (0.72-2.40), p=.37 |  | <5^1^ (1%) | 0.21 (0.05-0.92), p=.04 |  | | 5 (2%) | 0.81 (0.26-2.13), p=.59 |
|  |  |  |  |  |  |  |  |  | |  |  |
| 1. **Uptake of past 30-day smoking, vaping, or both among those who neither smoked nor vaped in the past 30 days at baseline (n=2,375):** | | | | | | | | | | | |
|  | No past 30-day smoking or vaping (n=2,182; ref) |  | Initiation of past 30-day smoking only (n=62) | |  | Initiation of past 30-day vaping only (n=88) | |  | Initiation of both (n=43) | | |
| **Perception that vaping is less harmful than smoking** | n (%) |  | n (%) | aRRR (95% CI), p |  | n (%) | aRRR (95% CI), p |  | | n (%) | aRRR (95% CI), p |
| Yes | 935 (91%) |  | 31 (3%) | Ref |  | 42 (4%) | Ref |  | | 21 (2%) | Ref |
| No | 796 (93%) |  | 19 (2%) | 0.71 (0.39-1.27), p=.25 |  | 27 (3%) | 0.69 (0.42-1.13), p=.14 |  | | 14 (2%) | 0.83 (0.41-1.68), p=.61 |
| Don’t know | 451 (92%) |  | 12 (2%) | 0.75 (0.38-1.49), p=.42 |  | 19 (4%) | 0.88 (0.50-1.54), p=.65 |  | | 8 (2%) | 0.68 (0.29-1.59), p=.38 |

aRRR=adjusted Relative Risk Ratio (Adjusted for baseline questionnaire completed, sex assigned at birth, race/ethnicity, unemployed/not in education at age 22, mother/partner occupation, mother smoked during first 3 months of pregnancy); 95% CI=95% Confidence Interval.

^1^ Cell counts less than 5 (including zero) are reported as <5 in line with ALSPAC’s requirements <https://www.bristol.ac.uk/media-library/sites/alspac/documents/alspac-publications-checklist.pdf>.
